# Limited incremental prognostic value of dyadic self- and informant-reported everyday cognition for incident MCI or Alzheimer’s disease dementia in ADNI: an exploratory prognostic modelling study

**DOI:** 10.21203/rs.3.rs-10884877/v2

**Published:** 2026-09-08

**Authors:** Wan Kiu Winkey Cheng

**Affiliations:** 1 Department of Biomedical Engineering, College of Biomedicine, City University of Hong Kong, Kowloon Tong, Hong Kong SAR, China

**Keywords:** subjective cognitive decline, dyadic informant report, ECog, prognostic model, ADNI

## Abstract

**Background:**

Subjective cognitive decline (SCD) measures, including self- and informant-reported concern, are candidate prognostic markers for Alzheimer’s disease (AD), but their incremental value over conventional biomarkers is uncertain. We evaluated, in an internally cross-validated ADNI sample, whether a dyadic mean and self–informant gap of baseline Everyday Cognition (ECog) total scores improve prediction of first recorded clinical diagnosis of MCI or AD dementia beyond the prespecified conventional predictors.

**Methods:**

We included 1,762 cognitively normal at-risk ADNI participants (events=288: 261 MCI, 27 AD dementia). Elastic-net Cox models (M2 conventional-predictor, M3 conventional-predictor + dyadic mean, M4 conventional-predictor + mean + gap) were fit using nested cross-validation with fold-wise imputation and standardization.

**Results:**

M2 C-index 0.682, M3 0.692, M4 0.687. Adding the dyadic mean yielded an estimated ΔC of 0.010 (95% CI −0.003 to 0.024), which was attenuated to ΔC = 0.002 (95% CI −0.013 to 0.018) after including landmark-year protocol-era candidate variables. The gap did not improve discrimination beyond the dyadic mean. In an exploratory biomarker-naive gatekeeping comparison, adding dyadic ECog to a clinical baseline of age, sex, education, and MMSE raised the C-index from 0.635 to 0.678 (ΔC = 0.044, 95% CI 0.023 to 0.064), nearly matching the biomarker-rich model (ΔC = 0.004, 95% CI −0.026 to 0.033 for M2 versus the gatekeeping model).

**Conclusions:**

In this internally validated ADNI cohort, a baseline dyadic ECog mean provided at most a small and statistically uncertain increment in prognostic discrimination beyond conventional predictors (ΔC = 0.010, 95% CI −0.003 to 0.024), which was largely attenuated after including protocol-era and report-availability candidate variables (ΔC = 0.002). The self–informant gap provided no additional discrimination. Dyadic ECog may be better positioned as a low-cost gatekeeper in biomarker-naive settings than as an add-on to biomarker-rich models; neither role is established without external validation.

## Introduction

Cognitively normal older adults at risk of subsequent MCI/AD dementia are an important target for prognostic modelling and trial enrichment. Self-reported and informant-reported cognitive concerns are associated with subsequent clinical progression in observational cohorts and meta-analyses [1–11], but association does not establish incremental value beyond an information-rich prognostic model. The stricter question is whether a single baseline dyadic summary improves discrimination after conventional demographic, cognitive, genetic, amyloid, and neurodegeneration predictors are already available. The dyadic mean and self–informant gap may capture perceived overall severity and divergence in awareness [4,5,7,12,13], but their added value is likely to depend on disease stage, measurement form, relationship context, and the process determining report availability. The most plausible real-world role for such reports may be as inexpensive gatekeepers within tiered screening pathways—identifying who warrants downstream biomarker testing—rather than as additions to already biomarker-rich models. At the same time, report availability in longitudinal cohorts is often protocol-dependent, so apparent prognostic signals attributed to subjective-report instruments may partly encode assessment-era structure rather than individual-level biology. Few prior studies have separated association from incremental discrimination while controlling leakage and accounting for established biomarker predictors.

The Everyday Cognition (ECog) scale is a structured self- and informant-report instrument that samples day-to-day cognitive difficulties across multiple domains [3,11]. In ADNI, the 39-item full form and the 12-item short form are each scored as the mean of completed items, with higher totals indicating greater subjective difficulty [3,11]. Because different ADNI phases administered different forms, the dyadic score represents an operational composite across phases; we did not assume formal psychometric measurement invariance across forms and analyzed the ADNI-supplied total scores as provided. While subjective cognitive decline has been repeatedly associated with longitudinal progression [2,4,12–14], whether routine dyadic questionnaires provide actionable incremental discrimination over standard conventional predictors (APOE4, Centiloid amyloid, and hippocampal volume) is a critical question for resource allocation in Alzheimer’s disease research cohorts and trial-enrichment workflows [6,7,10,15–18].

We therefore conducted an exploratory prognostic modelling study in the Alzheimer’s Disease Neuroimaging Initiative (ADNI) to establish a rigorous, leakage-free benchmark for whether baseline dyadic ECog scores improve prediction of progression from cognitively normal status to first recorded clinical diagnosis of MCI or AD dementia beyond a prespecified conventional-predictor model. We specified the model hierarchy, primary contrast, and fold-wise workflow before the final rerun; this included four nested Cox elastic-net models and a primary contrast (M3 versus M2) evaluating the incremental value of the dyadic mean, alongside additional robustness analyses in complete dyads, alternative endpoints, and interval-censoring placements. This work follows prognostic-modelling reporting standards (TRIPOD) and computational reproducibility practices.

## Methods

### Study design and participants

We included 1,762 ADNI participants who were cognitively normal and had no prevalent MCI or AD dementia diagnosis at the landmark visit (the earliest visit classified as cognitively normal; the landmark visit was typically the ADNI screening or baseline visit at which the participant was first classified as cognitively normal). At the landmark visit, participants were required to have a clinical diagnosis of cognitively normal (CN), a global CDR score of 0, and an MMSE score of at least 24; if CDR or MMSE was missing, the diagnosis was required to be explicitly CN and the remaining cognitive record was required to be consistent with normal cognition. The primary endpoint was the first recorded clinical diagnosis of MCI or AD dementia after the landmark, identified from ADNI longitudinal diagnostic summary records (DIAGNOSIS/DXSUM, tracking transition from CN to incident MCI or dementia). Events were 261 incident MCI and 27 incident AD dementia; 1,474 participants were censored. Censoring occurred at the last available visit, withdrawal, or death. Death was treated as right-censoring in the primary Cox analysis. Formal competing-risk survival models were not fitted; therefore, estimated associations may be subject to bias if non-dementia death was informative, which should be considered when translating findings to older populations with higher competing mortality. The full-cohort analysis addressed a pragmatic estimand: the incremental discrimination obtained when dyadic ECog information is incorporated into an ADNI-like workflow in which report availability is protocol-dependent. The complete-dyad analysis addressed a distinct conditional estimand: the incremental discrimination of the dyadic mean among participants for whom both self- and informant-reported ECog assessments were observed. These analyses were interpreted separately because dyad availability was strongly associated with assessment era, multimodal data acquisition, and observed follow-up (Supplemental Table 1b). A separate CDR-only sensitivity endpoint was defined as first CDR global score ≥0.5 in the absence of a recorded clinical MCI/AD event; baseline CDR was required to be 0 and a single-visit crossing was treated as the event. The three interval-censoring placements were deterministic event-time assignments (first event, midpoint, and last-known-normal) used as sensitivity analyses [19]. ADNI is a longitudinal multi-site observational study launched in 2003 to develop and validate imaging, biomarker, and clinical measures for MCI and AD [20–23]. ADNI data files used for this analysis were downloaded on 31 January 2026; the latest usable follow-up information in the downloaded snapshot corresponded to 3 November 2025.

Data used in the preparation of this article were obtained from the Alzheimer’s Disease Neuroimaging Initiative (ADNI) database (adni.loni.usc.edu). ADNI was launched in 2003 as a public-private partnership, led by Principal Investigator Michael W. Weiner, MD. The primary goal of ADNI has been to test whether serial magnetic resonance imaging, positron emission tomography, other biological markers, and clinical and neuropsychological assessments can be combined to measure the progression of mild cognitive impairment and early Alzheimer’s disease. For up-to-date information, see www.adni-info.org.

### Predictors

Model M0 (clinical baseline) included age, sex (binary, female=1 if self-reported sex was female), education, and MMSE. Model M2 (conventional-predictor model) extended the clinical baseline with APOE4 carrier status (coded 1 if at least one 4 allele was present and 0 otherwise), continuous Centiloid, hippocampal volume, and prespecified missingness indicators for these three variables, drawing on established AD genetic, amyloid, and neurodegeneration markers [15–18,24]. Hippocampal volume was the ADNI-derived total bilateral (left+right combined) hippocampal volume in mm^3^; the bilateral combined value was used as the primary measure and left-only volumes were not substituted, with no additional intracranial-volume adjustment. Centiloid was the primary continuous amyloid measure. Binary amyloid positivity was not included in the primary M2 model.

Primary ECog self-report and informant-report total scores were the ADNI-derived, precomputed mean of completed items on the 39-item full form (ADNI2/3/GO) or the 12-item short form (ADNI4), with each item scored on a 1-4 Likert scale and higher total scores indicating greater concern about everyday cognitive functioning [3,11]. We used the ADNI-derived total scores as supplied, did not rederive item-level means, and treated a missing total as missing when ADNI did not provide a valid score for that form. The dyadic mean and gap were calculated only when both self and informant total scores were available. When the dyadic mean or gap was unavailable, the score was median-imputed within each outer-training fold and was accompanied by the dyad-availability indicator; no single-informant value was substituted for the missing dyadic mean or gap. No separate missingness indicators for the dyadic mean or gap were created; dyad availability was represented solely by the dyad-availability indicator. The linear gap (informant minus self) was prespecified as the primary discrepancy measure for parsimony and interpretability; non-linear or categorical discrepancy profiles (for example, self-over-reporting versus informant-predominant patterns) were not included in the prespecified model hierarchy and are discussed as future directions.

Missingness in the baseline predictors, including age, education, MMSE, APOE4, Centiloid, hippocampal volume, and dyadic ECog, was not random: it was strongly linked to ADNI study phase, protocol evolution, and scan availability. To evaluate model discrimination under pragmatic dataset conditions without discard-induced power loss, we used fold-wise median imputation (calculated strictly from available observations within each outer-training fold and projected to outer-test data) accompanied by candidate missingness indicators for each missing baseline predictor; the dyadic mean and gap used the dyad-availability indicator rather than separate score-missingness indicators. These indicators were available to the elastic-net models and could be shrunk toward zero during penalized fitting. The missingness-indicator method allows the model to capture pragmatic cohort patterns; this choice is defensible here because the study objective is out-of-fold discrimination under the observed measurement process, not unbiased estimation of covariate–outcome etiological associations [25–27]. The resulting coefficients must therefore be interpreted as empirical adjustment weights rather than unbiased biological or etiological parameters [25–27]. To provide a conditional observed-dyad benchmark that did not require imputation of the dyadic ECog score or inclusion of a dyad-availability indicator, a complete-dyad analysis (both self and informant observed, n=477, events=88) was conducted as an additional conditional analysis. Missingness in non-ECog predictors was handled using the same fold-wise procedures as in the primary analysis.

### Statistical analysis

We fit elastic-net Cox survival models with l1_ratio=0.5 [28–31]. The mixing parameter l1_ratio=0.5 was prespecified to balance the sparse variable selection of Lasso with the grouping property of Ridge regularization under collinearity among demographic, cognitive, and biomarker predictors. Lambda was selected from {0.001, 0.005, 0.01, 0.05, 0.1} by inner 3-fold cross-validation. All preprocessing (imputation, standardization, residualisation where used, and lambda selection) was fit inside each outer-training fold only. Outer test participants were never used during model fitting. We used Harrell’s concordance index (scikit-survival concordance_index_censored) on pooled out-of-fold (OOF) linear predictions to estimate discrimination [32–34]. The primary objective was to estimate the incremental prognostic discrimination associated with the dyadic predictors under the primary analysis pipeline. Because the model was not intended for deployment as an absolute-risk calculator, and because the dyad-availability indicator exhibited non-proportional hazards, post-hoc predicted probabilities were not treated as clinically calibrated risks, and decision-curve analysis was not performed.

A nested cross-validation structure was essential to avoid information leakage. The outer 5-fold loop, stratified by the primary event indicator, provided an approximately unbiased estimate of discrimination in the same population; the inner 3-fold loop selected the penalty parameter without touching the outer test fold [35,36]. All variable-specific standardization, imputation, and residualisation parameters were estimated on the outer-training folds and applied to the corresponding test fold. This means that the reported C-index is a conditional out-of-fold performance estimate, not an apparent or resubstitution estimate. Because the model was developed and evaluated within the same ADNI cohort, the C-index may still be optimistic relative to an independent external sample [37,38].

Paired bootstrap contrasts (1,000 replicates) compared OOF C-index values between models. Bootstrap confidence intervals were calculated by resampling participants’ paired out-of-fold predictions, taking the 2.5th and 97.5th percentiles of the bootstrap distribution, and therefore quantify uncertainty conditional on the fitted cross-validation pipeline; they do not fully capture model-selection uncertainty from the inner cross-validation loop.

### Proportional-hazards and sensitivity diagnostics

We evaluated the proportional-hazards assumption with Schoenfeld residual tests on rank-transformed event times for the primary M3 and M4 Cox models, plus variable-specific Schoenfeld tests [39,40]. Rank transformation was used only for the diagnostic test statistic and did not alter the original time scale in the primary elastic-net Cox models. These diagnostics were performed in descriptive full-sample Cox models that used the same predictor specification, imputation, and coding as the primary elastic-net models; they were not used to retune or refit the nested cross-validation elastic-net models. The resulting p-values are exploratory postfit checks, not a formal validation that the penalized primary models satisfy proportional hazards; because the full-sample coefficients differ from the fold-specific penalized estimates, the diagnostics provide context rather than direct proof. Because the diagnostics are exploratory and because predictors such as dyad availability showed evidence of non-proportionality, the Cox models are used here primarily to compare relative prognostic discrimination under the analysis pipeline and should not be interpreted as providing stable time-invariant hazard ratios or clinically calibrated absolute risks. Sensitivity analyses were: residualised versus raw dyadic scores; a missingness-only comparison (M2 plus the dyad-availability indicator versus M3); a complete-dyad subset (both self and informant observed, with models re-estimated de novo); minimum follow-up restrictions (≥0.5 years and ≥2.0 years); a protocol-era candidate-variable sensitivity model in which landmark year was represented by categorical bands (2005–2010, 2011–2014, 2015–2018, 2019–2022, and 2023–2025); three interval-censoring placements of the event time (first-event, midpoint, and last-known-normal) [19]; these deterministic placements were retained because they preserve an identical penalized pipeline, fixed folds, and paired out-of-fold contrasts across endpoint definitions, whereas non-parametric interval-censored estimators would have required a different modelling framework; the CDR-only endpoint; an MRI availability audit; an exploratory biomarker-naive gatekeeping comparison (clinical baseline plus dyadic ECog versus the conventional-predictor model); and a repeated nested-cross-validation split-stability analysis using 10 independently generated stratified 5-fold partitions. The repeated analysis used identical outer folds for M2, M3, and M4 within each repetition and repeated inner 3-fold penalty selection; repeat-level contrasts were summarized descriptively rather than treated as an additional confirmatory hypothesis test.

## Results

### Cohort characteristics and missingness

Follow-up characteristics for the primary ADNI sample were: median time to event or censoring 0.96 years (IQR 0.00–3.62; range 0.00–13.31); median event time 2.76 years (IQR 1.03–6.27); median censoring time 0.34 years (IQR 0.00–2.81); total person-years 3418.5. A large proportion of participants were censored early, reflecting the ADNI visit schedule and the reality that many cognitively normal participants had limited longitudinal follow-up.

Dyadic ECog data were available for approximately 27% of the cohort, reflecting a high rate of missing self- or informant-reported cognitive concern. APOE4 carrier status was available for 70% of participants; amyloid and hippocampal imaging were available for approximately 37% and 35%, respectively.

### Primary model discrimination

The primary contrast (M3 versus M2) showed only a small raw increment in discrimination (ΔC = 0.010, 95% CI −0.003 to 0.024). The confidence interval was compatible with no incremental discrimination, so the dyadic mean did not provide robust evidence of added prognostic value. Adding the self–informant gap (M4 versus M3) produced essentially no change (ΔC = −0.005, 95% CI −0.010 to 0.000).

### Sensitivity and diagnostic analyses

Dyadic data were available for only about 27% of participants, so any apparent incremental signal from the dyadic mean may be partly entangled with whether a dyadic report is available. Dyad availability was associated with non-proportional hazards and was strongly patterned by study-protocol evolution and assessment era. In an exploratory, unadjusted sensitivity comparison, the dyadic-mean model (M3) yielded a slightly higher C-index than the availability-only model (M2 plus the dyad-availability indicator; C-index 0.692 versus 0.678; ΔC = 0.013, 95% CI 0.001 to 0.028). In the complete-dyad subset the mean increment was numerically larger (ΔC = 0.028, 95% CI −0.006 to 0.064), but this selected analysis included only 88 events and was not treated as confirmatory. Minimum follow-up sensitivity analyses restricted to participants with at least 0.5 years of follow-up (n=979, events=288; M2 0.685 vs M3 0.696; ΔC = 0.011, 95% CI −0.002 to 0.025) and at least 2.0 years of follow-up (n=816, events=288; M2 0.706 vs M3 0.715; ΔC = 0.008, 95% CI −0.004 to 0.021) yielded incremental discrimination estimates consistent with the full primary cohort. Furthermore, when models included landmark-year bands as protocol-era candidate variables (n=1,762, events=288), the dyadic-mean increment was attenuated to ΔC = 0.002 (95% CI −0.013 to 0.018; M2 0.697 vs M3 0.699; Table 3 and Supplemental Table 2), indicating that the small raw increment was not robust to inclusion of landmark-year-band candidate variables, which serve as a proxy for intertwined protocol-era, follow-up, and assessment-process differences. Across 10 independent repetitions of nested 5-fold cross-validation, the median M3–M2 increment was 0.0109 (IQR 0.0072–0.0132; range 0.0052–0.0151), indicating that the small magnitude of the raw increment was relatively stable across the examined partition splits (Supplemental Table 8; Supplemental Figure 4). These repetitions reused the same underlying ADNI participants and therefore assessed partition sensitivity rather than independent external reproducibility. The global proportional-hazards test for the primary M3 model did not indicate an overall departure from proportionality (χ^2^(15) = 21.43, p = 0.123). However, the dyad-availability indicator showed evidence of non-proportionality (p = 0.001), whereas the dyadic mean (p = 0.222) and the dyadic gap (p = 0.998) did not. Full Schoenfeld test results are reported in Supplemental Table 3. Finally, in an exploratory biomarker-naive gatekeeping comparison on the same fixed folds, adding the dyadic ECog mean and the dyad-availability indicator to the clinical baseline model (M0: age, sex, education, MMSE) raised the out-of-fold C-index from 0.635 to 0.678 (ΔC = 0.044, 95% CI 0.023 to 0.064), nearly matching the conventional biomarker-rich model (M2 0.682; ΔC = 0.004, 95% CI −0.026 to 0.033 for M2 versus the gatekeeping model; Table 3). Within the completedyad subset, where the dyad-availability indicator is constant and drops out of the model, the corresponding increment over M0 was ΔC = 0.028 (95% CI −0.021 to 0.082; M0 0.646, gatekeeping model 0.674, n=477, events=88), suggesting that part of the gatekeeping signal reflects ECog score content rather than availability alone, although the estimate is imprecise.

## Discussion

In a leakage-free, internally cross-validated ADNI sample, the baseline dyadic mean of self- and informant-reported ECog scores provided at most a small and statistically inconclusive increment in out-of-fold C-index over the prespecified conventional-predictor model (0.692 versus 0.682; ΔC = 0.010, 95% CI −0.003 to 0.024), and adding the self–informant gap provided no further discrimination (0.687; ΔC = −0.005, 95% CI −0.010 to 0.000). Although self- and informant-reported concerns have been associated with later decline in prior studies, association does not guarantee incremental utility after established predictors are included. In a biomarker-rich setting where amyloid PET and hippocampal volume are already acquired, these established markers capture the majority of prognostic variance, leaving little residual signal for a static baseline questionnaire to add. However, this information redundancy in biomarker-characterized cohorts does not preclude the potential utility of dyadic reports in biomarker-naive or primary triage contexts, where questionnaires could serve as accessible gatekeepers to determine who warrants downstream biomarker testing [1,6–10]. Consistent with that positioning, an exploratory biomarker-naive comparison found that adding dyadic ECog to a clinical baseline of age, sex, education, and MMSE raised out-of-fold discrimination from 0.635 to 0.678 (ΔC = 0.044, 95% CI 0.023 to 0.064), nearly matching the conventional biomarker-rich model; this estimate is nevertheless exploratory, because the dyad-availability indicator it incorporates is itself protocol-linked. Within the complete-dyad subset, where the availability indicator is constant and cannot carry signal, the increment over M0 was ΔC = 0.028 (95% CI −0.021 to 0.082), indicating that part of the gatekeeping effect reflects ECog score content while the remainder may be entangled with availability; this gatekeeping analysis is a proof-of-concept that requires validation in cohorts where dyadic questionnaires are administered universally rather than under protocol-dependent schedules. More broadly, these findings carry a caution for the observational literature: apparent prognostic signals attributed to subjective-report instruments may partly encode protocol-era and report-availability structure, so incremental claims should be stress-tested against such proxies before being attributed to the instrument itself.

Dyadic cognitive reports may not contribute uniformly across the Alzheimer’s disease continuum. Prior work suggests that the relative informativeness of self- and informant-reported concern can vary with disease stage, objective impairment, and the direction of dyadic discrepancy [1,6,8–10,41–44]. In particular, self–informant discrepancy often exhibits bidirectional and non-linear patterns (such as heightened self-concern or hypernosognosia in early subjective decline versus informant-predominant concern or anosognosia approaching clinical transition). A simple linear subtraction (gap = informant - self) may mask these opposing directional effects, suggesting that future research should explore non-linear feature engineering or categorical thresholding (e.g., self-over-reporters versus anosognosic dyads). Under the observed ADNI assessment process, a simple static linear summary does not add decisive prognostic power beyond conventional markers.

Several mechanisms explain the modest incremental value of the dyadic score. First, dyadic ECog was available for only about 27% of the total cohort, reflecting protocol-driven missingness across ADNI phases [25,27]. In our exploratory sensitivity comparison, an availability-only model (M2 plus the dyad-availability indicator) achieved a C-index of 0.678 compared to 0.692 for M3. In ADNI, dyad availability reflects a combination of study protocol evolution (routine administration in ADNI2/3/GO versus historical phases), study-partner availability, participant engagement, and multimodal visit completion (Supplemental Table 1b). As detailed in Supplemental Table 1b, participants with available dyads (n=477) had markedly higher biomarker acquisition (Centiloid available in 87.4% vs 17.6%; hippocampal volume in 86.0% vs 16.0%) and longer follow-up (median 3.86 vs 0.22 years) than those with unavailable dyads (n=1,285). These operational mechanisms cannot be fully disentangled from individual-level social or clinical factors. In an exploratory comparison, M3 showed a small residual discrimination increment beyond the availability-only model (ΔC = 0.013, 95% CI 0.001 to 0.028) [4,7]; this estimate requires cautious interpretation because the comparison remains vulnerable to protocol-linked selection, multiple analyses, and conditional bootstrap uncertainty. Across every robustness check performed—restricting to complete dyads, requiring minimum follow-up duration, including protocol-era candidate variables, and repeating cross-validation splits—the increment attributable to baseline dyadic ECog remained small and, under protocol-era modeling, negligible.

Consequently, the conventional-predictor model should be interpreted as a pragmatic prediction model conditioned on the observed ADNI measurement process, rather than as a model quantifying the independent biological contribution of a uniformly measured biomarker panel. In the complete-dyad sensitivity subset where questionnaires were actively administered (n=477), the dyadic-mean increment was numerically larger (ΔC = 0.028), yet the sample size and event count (events=88) were restricted and the confidence intervals remained broad, reinforcing that static baseline dyadic scores do not yet provide robust standalone utility. The gap between this conditional estimate obtained under uniformly administered dyads (ΔC = 0.028) and the pragmatic full-cohort estimate obtained under protocol-dependent availability (ΔC = 0.010) illustrates how strongly the perceived promise of dyadic scores depends on the assessment scenario assumed.

A strength of this study is the explicitly specified analysis workflow and the fully fold-wise implementation with strict safeguards against leakage. By fitting all preprocessing and model selection within each outer-training fold and by using the same fixed cross-validation folds across sensitivity analyses, we aimed to obtain an internally consistent comparison between M2, M3 and M4. The pipeline is scripted end-to-end from a single entry point with fixed seeds and fixed cross-validation folds, and every reported statistic is source-keyed to a locked results registry, so each number in this manuscript can be traced to a specific generated output. A completed TRIPOD checklist and archived analysis code are provided to support independent verification and reproducibility [45,46].

The study has important limitations. First, it is a single-cohort, internally cross-validated analysis; the findings require external validation in independent cohorts before any clinical application [37,38]. Second, the high rate of missing dyadic data reflects historical cohort design; although addressed pragmatically via fold-wise missingness indicators and parallel complete-dyad analyses, selection mechanisms cannot be fully eliminated [25,27]. Third, the median overall follow-up was 0.96 years, with censored participants having a median follow-up of 0.34 years (events median 2.76 years); although early censoring was substantial, incremental discrimination was consistent in sensitivity analyses restricted to participants with at least 0.5 years of follow-up (ΔC = 0.011, 95% CI −0.002 to 0.025) and at least 2.0 years of follow-up (ΔC = 0.008, 95% CI −0.004 to 0.021; Table 3 and Supplemental Table 2). Fourth, we utilized ADNI-derived ECog totals across phases without assuming formal psychometric equivalence between the 39-item and 12-item versions. Fifth, because clinical diagnostic adjudication and functional staging in ADNI may conceptually overlap with subjective or informant-reported functional complaints, potential incorporation bias cannot be fully excluded; similarly, the CDR-based sensitivity endpoint incorporates informant input during semi-structured clinical assessment. Sixth, deterministic event-time placements were used for interval-censoring sensitivities rather than non-parametric interval-censored models [19]. Seventh, death was treated as right-censoring rather than in a formal competing-risk framework (for example, Fine–Gray subdistribution hazards); because non-dementia mortality preferentially removes older or frailer participants who may carry higher progression risk, informative competing mortality would generally attenuate the observed risk gradient, although the direction of any residual bias on the incremental C-index contrast is uncertain. Eighth, landmark-year bands are administrative proxy variables, not direct measurements of specific biological factors or isolated study protocols; therefore, attenuation in the landmark-year-band candidate-variable sensitivity analysis should be interpreted as sensitivity to temporally patterned assessment processes, rather than proof that protocol era causally generated the raw dyadic signal. These findings are most directly applicable to research cohorts with ADNI-like, protocol-dependent multimodal assessment, rather than to unselected community or routine memory-clinic populations. Finally, because the primary objective was comparative incremental discrimination under the primary analysis pipeline rather than developing a deployable absolute-risk tool, and because dyad availability exhibited non-proportional hazards, post-hoc predicted probabilities should not be interpreted as calibrated clinical absolute risks.

These results do not support using static baseline dyadic ECog totals as standalone or decisive prognostic tools in biomarker-characterized cognitively normal older adults. Future work should investigate longitudinal dyadic trajectories, non-linear or categorical discrepancy profiles (e.g., hypernosognosic versus anosognosic dyads), combination with scalable plasma biomarkers (such as p-tau217 or neurofilament light chain) within tiered multistage screening protocols before confirmatory PET or CSF testing, and prospective external validation in cohorts with harmonised dyadic instruments [6,38].

In conclusion, although self- and informant-reported cognitive concerns are associated with later decline in prior studies, a single baseline dyadic ECog summary did not provide robust incremental prognostic discrimination beyond conventional clinical, genetic, amyloid, and neuroimaging predictors in cognitively normal ADNI participants. The raw increment was small and uncertain, was sensitive to protocol-era candidate-variable inclusion, and the self–informant gap provided no additional discrimination. These findings do not support treating a static baseline dyadic ECog summary as a decisive prognostic tool without external validation in cohorts with harmonised assessment and follow-up procedures.

## Supplementary Material

This is a list of supplementary files associated with this preprint. Click to download.

- supplemental1.docx
- supplemental2scpublish.zip

## Figures and Tables

**Figure 1.**
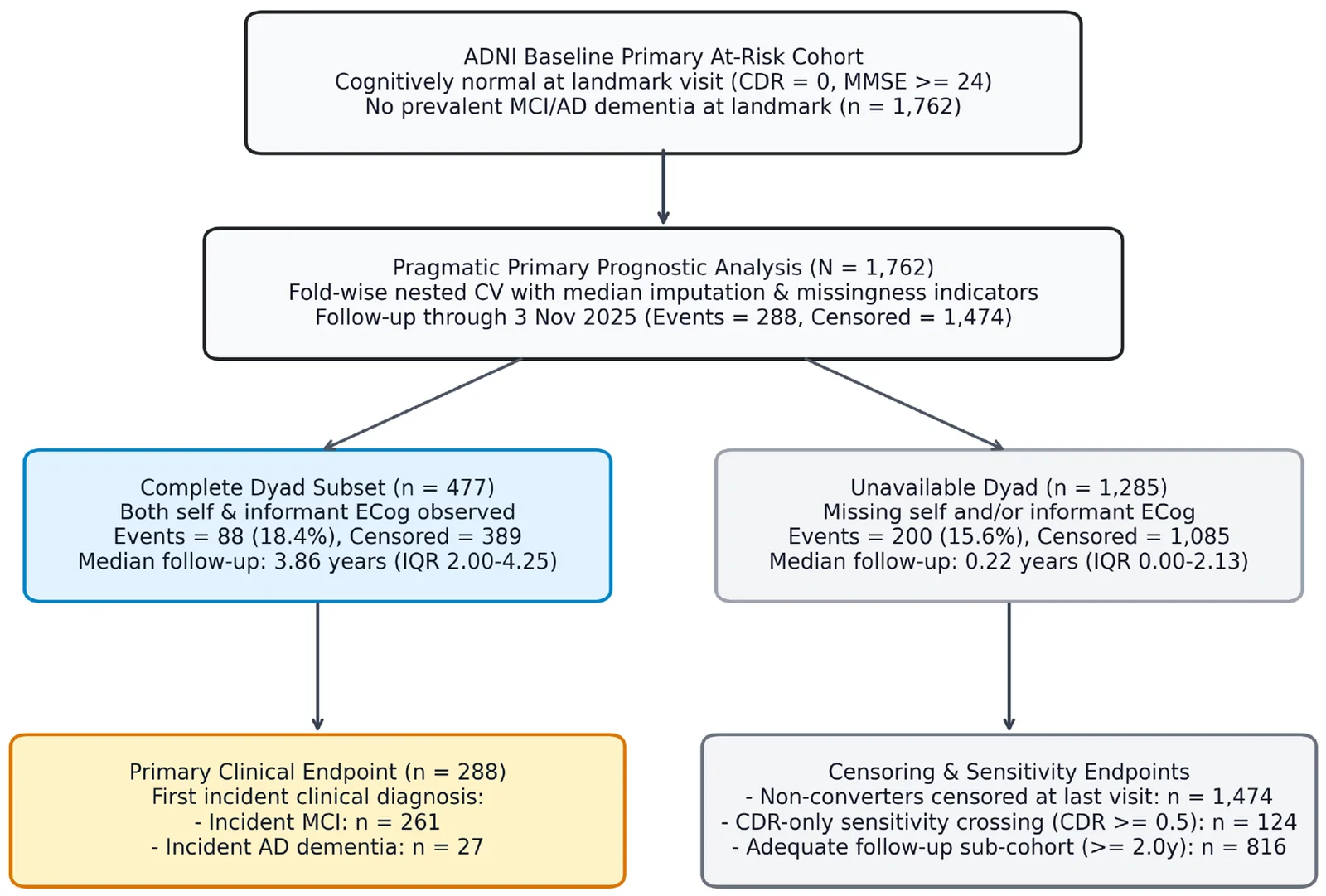
ADNI participant flow and estimand architecture. Schematic flowchart illustrating the full pragmatic at-risk cohort (N=1,762), the complete-dyad conditional subset (n=477), the primary clinical endpoint breakdown (n=288: 261 incident MCI, 27 incident AD dementia), and sensitivity endpoints.

**Figure 2.**
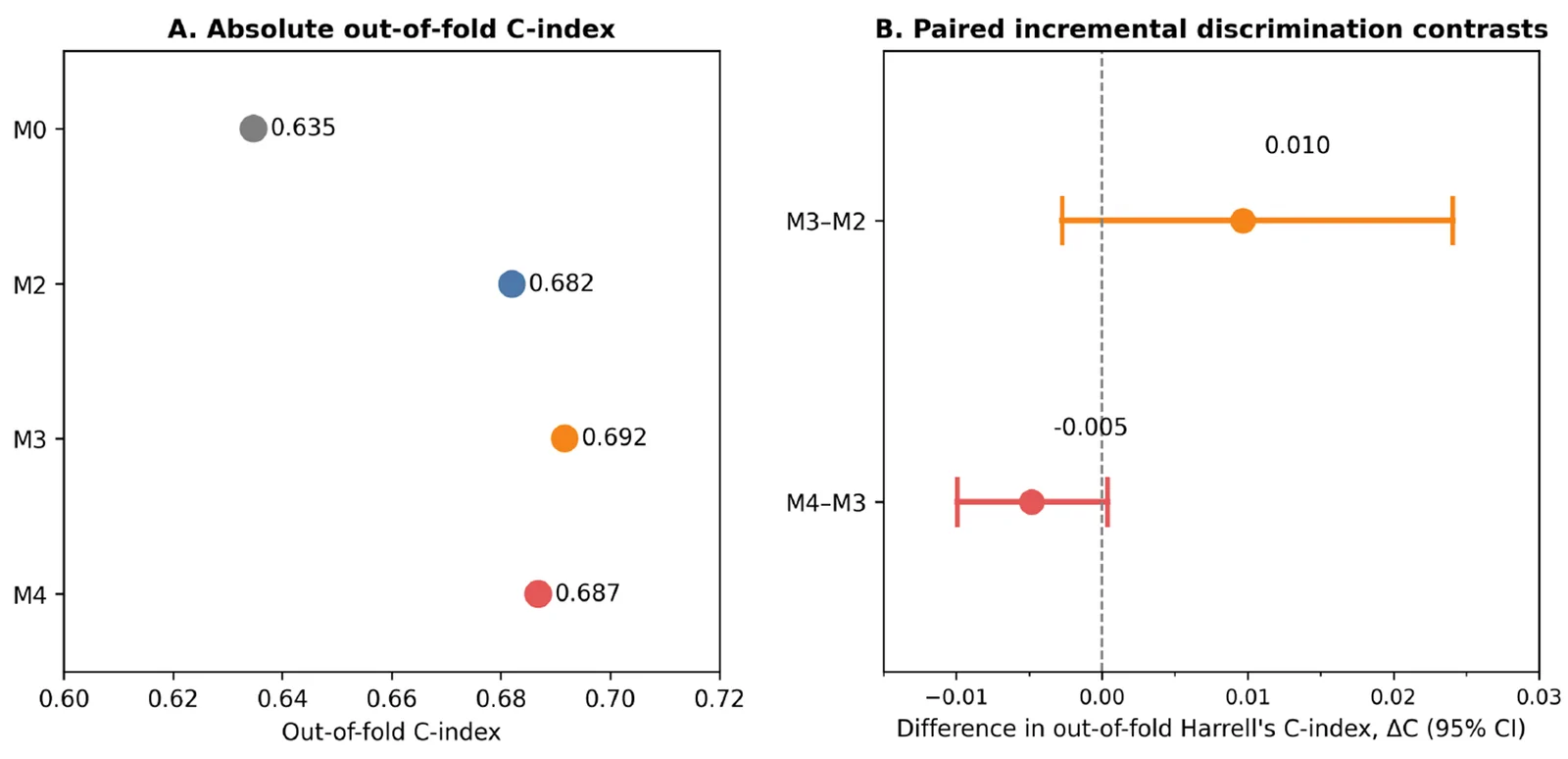
Out-of-fold discrimination across prespecified models and paired incremental discrimination contrasts. A, pooled out-of-fold Harrell’s C-index for the clinical baseline model (M0), conventional-predictor model (M2), conventional-predictor model plus dyadic mean ECog score (M3), and M3 plus the self-informant ECog gap (M4). B, paired bootstrap contrasts comparing the primary dyadic-mean contrast (M3-M2) and the secondary dyadic-gap contrast (M4-M3). Points indicate estimated differences in C-index and horizontal bars indicate percentile 95% confidence intervals from 1,000 paired bootstrap replicates. The dashed vertical line denotes no difference in discrimination. The M3-M2 contrast was small and statistically uncertain; adding the self-informant gap did not improve discrimination beyond M3.

**Table 1.** ADNI cohort characteristics and predictor missingness. N is the number of non-missing observations for each characteristic; percentages for categorical variables are computed within the non-missing N. Continuous variables: mean (SD). Categorical variables: n (%). Percent missing refers to the proportion missing in the full n=1,762 cohort. High missingness for ECog, amyloid, and MRI reflects ADNI multi-phase recruitment and variable collection protocols rather than a single-clinic cohort with uniform attrition.

Characteristic	N	Summary	% missing
Age, years	1761	71.71 (7.91)	0.06
Education, years	1759	16.45 (2.54)	0.17
MMSE	1757	28.97 (1.20)	0.28
Female, n (%)	1761	1046 (59.4)	0.06
APOE4 carrier, n (%)	1236	409 (33.1)	29.85
Centiloid	643	22.91 (39.48)	63.51
Amyloid positive, n (%)	643	213 (33.1)	63.51
Hippocampal volume	616	7781.54 (859.77)	65.04
ECog self total	485	1.43 (0.42)	72.47
ECog informant total	479	1.21 (0.29)	72.81
ECog dyadic mean	477	1.32 (0.29)	72.93
ECog dyadic gap	477	−0.22 (0.43)	72.93

**Table 2.** Out-of-fold C-index for the locked ADNI models.

Model	N	Events	C-index
M0	1762	288	0.635
M2	1762	288	0.682
M3	1762	288	0.692
M4	1762	288	0.687
Contrast	ΔC	95% CI
M3 – M2	0.010	−0.003 to 0.024
M4 – M3	−0.005	−0.010 to 0.000

Paired bootstrap contrasts. CIs were conditional on the fitted out-of-fold pipeline and do not incorporate the full uncertainty of repeated tuning and model selection.

**Table 3.** Sensitivity and diagnostic analyses. Minimum-follow-up analyses included participants with at least the specified duration of observed follow-up or an observed primary event before that threshold. The primary model hierarchy and M3–M2 contrast were prespecified; sensitivity analyses evaluating follow-up duration, protocol era, and dyad availability were conducted to test the robustness of the primary estimates. The biomarker-naive gatekeeping rows compare the clinical baseline (M0) against M0 plus the dyadic ECog mean and dyad-availability indicator; these were exploratory analyses evaluating the questionnaire as a low-cost front-end screen. In the complete-dyad gatekeeping row the availability indicator is constant, so the contrast reflects ECog score content alone.

Sensitivity / analysis	N	Events	Reference C-index	M3 C-index	M3–Ref ΔC (95% CI)
Primary (raw, full sample)	1762	288	0.682	0.692	0.010 (−0.003 to 0.024)
M2 + dyad availability vs M3	1762	288	0.678	0.692	0.013 (0.001 to 0.028)
Biomarker-naive gatekeeping (M0 + dyadic ECog vs M0)	1762	288	0.635	0.678	0.044 (0.023 to 0.064)
Biomarker-naive gatekeeping, complete dyads (M0 + ECog mean vs M0)	477	88	0.646	0.674	0.028 (−0.021 to 0.082)
Residualised dyadic scores	1762	288	0.682	0.691	0.009 (−0.003 to 0.024)
Complete-dyad subset	477	88	0.663	0.691	0.028 (−0.006 to 0.064)
Minimum follow-up ≥0.5 years	979	288	0.685	0.696	0.011 (−0.002 to 0.025)
Minimum follow-up ≥2.0 years	816	288	0.706	0.715	0.008 (−0.004 to 0.021)
Protocol-era (landmark-year band) candidate variables	1762	288	0.697	0.699	0.002 (−0.013 to 0.018)
Interval-censoring: first-event endpoint	1762	288	0.682	0.692	0.010 (−0.003 to 0.024)
Interval-censoring: midpoint	1762	288	0.689	0.7	0.011 (−0.001 to 0.024)
Interval-censoring: last-known-normal endpoint	1762	288	0.748	0.752	0.003 (−0.006 to 0.013)
CDR-only endpoint (first CDR global ≥0.5)	1762	124	0.657	0.681	0.024 (−0.008 to 0.061)

## Data Availability

ADNI data are available through the ADNI Data Sharing Initiative (adni.loni.usc.edu), subject to approved data-use agreements. Analysis code, the analysis configuration, locked output manifests, and the final results registry for the ADNI analyses are archived at the Zenodo repository https://doi.org/10.5281/zenodo.22140401 and are publicly accessible. The Zenodo archive does not contain raw ADNI individual-level data; it provides the pipeline and non-data-derived outputs needed to reproduce the analysis in conjunction with ADNI data obtained under the required data-use agreement.
